# Faculty-Wide Peer-Support Program During the COVID-19 Pandemic: Design and Preliminary Results

**DOI:** 10.2196/37527

**Published:** 2023-03-02

**Authors:** Jenny J W Liu, P Andrea Lum, Laura Foxcroft, Rod Lim, J Don Richardson

**Affiliations:** 1 Schulich School of Medicine & Dentistry Western University London, ON Canada; 2 MacDonald Franklin OSI Research Centre Parkwood Institute Research London, ON Canada

**Keywords:** physician, doctor, medical professional, health care professional, peer support, burnout, mental distress, stress, work, job, peer leadership, mental health, occupational health, COVID-19

## Abstract

**Background:**

Physicians experience higher rates of burnout relative to the general population. Concerns of confidentiality, stigma, and professional identities as health care providers act as barriers to seeking and receiving appropriate support. In the context of the COVID-19 pandemic, factors that contribute to burnout and barriers to seeking support have been amplified, elevating the overall risks of mental distress and burnout for physicians.

**Objective:**

This paper aimed to describe the rapid development and implementation of a peer support program within a health care organization located in London, Ontario, Canada.

**Methods:**

A peer support program leveraging existing infrastructures within the health care organization was developed and launched in April 2020. The “Peers for Peers” program drew from the work of Shapiro and Galowitz in identifying key components within hospital settings that contributed to burnout. The program design was derived from a combination of the peer support frameworks from the Airline Pilot Assistance Program and the Canadian Patient Safety Institute.

**Results:**

Data gathered over 2 waves of peer leadership training and program evaluations highlighted a diversity of topics covered through the peer support program. Further, enrollment continued to increase in size and scope over the 2 waves of program deployments into 2023.

**Conclusions:**

Findings suggest that the peer support program is acceptable to physicians and can be easily and feasibly implemented within a health care organization. The structured program development and implementation can be adopted by other organizations in support of emerging needs and challenges.

## Introduction

### Background

Physicians experience a multitude of stressors and challenges associated with their day-to-day work. In addition to the demands of care provision, physicians also provide supervision, take on leadership and administrative duties, and act as educators in various capacities. Paralleling the multitude of stress experienced, physicians also report higher levels of burnout, depression, suicidal ideation, and mental distress relative to the general public [1]. For many physicians, these struggles are experienced in isolation. Research has documented that up to 35% to 50% of physicians do not disclose their health concerns, seek the support, and/or access resources when in need [2-5]. Instead, the juxtaposition of seeking support while providing health care to others creates a unique level of stigma for physicians that act as barriers to seeking help [2-7]. Indeed, this stigma is often referred to as a type of “silent suffering” among physicians [5]. Further, concerns with disclosure and documentation of individual health status are tied to fears of this information being used by regulatory bodies to impact and/or restrict licensure [3,5]. Together, they contribute to unmet mental health needs, the lack of awareness of available services, and the underutilization of available programs [8].

The COVID-19 pandemic amplified much of these challenges. Physicians experienced increased demands placed upon them at work, whereas the availability of support and resources decreased systematically within the health care system [7,9]. Further, research conducted with physicians during the pandemic reported elevated reports of moral distress and injury, such as having to make difficult decisions regarding delivery of lifesaving treatments, having to provide care despite inadequate personal protective equipment, and struggling with systemic and ethical issues that may conflict with one’s values and beliefs [10-12]. These conditions led to the deterioration of mental health and well-being of physicians. Emerging research indicate that mental health concerns and substance use among physicians have increased by as much as 27% during the pandemic [13]. However, solutions providing support must take into consideration the barriers to health care reported by physicians, such as overcoming issues of confidentiality and stigma. Taken together, the urgent demand for mental health support for physicians during the pandemic must be met with solutions that are sensitive to the special circumstances and barriers faced by physicians.

### Study Aims

Following observations of mental distress in physicians, the decanal office at Western University Schulich School of Medicine and Dentistry developed a peer support program, named Peers for Peers (P4P). The program sought to provide support to physicians across diverse departments and clinical divisions within the hospital network. This paper aimed to outline the process of development and highlight initial findings from program evaluations on the reach of the P4P program.

## Methods

### Ethical Considerations

Findings of this paper were conducted as part of a program evaluation for the P4P program, and thus was exempt from obtaining human subject research ethics review. Information collected were anonymous. No identifying information were collected as part of the program evaluation.

### Program Development

The development of the P4P program was led by faculty members within the Schulich School of Medicine and Dentistry following recommendations in the literature for providing support to clinicians [14]. Specifically, program foundations drew from the work of Shapiro and Galowitz [14] in identifying key components within hospital settings that contributed to burnout within clinicians, such as work burden and resource constraints. In addition, the program design was built upon the peer support frameworks from the Airline Pilot Assistance Program [15] and the Canadian Patient Safety Institute [16]. Specifically, the emphasis was placed on the recognition of internal cultures within medicine and leveraging existing peer leaders and expertise within the larger infrastructure.

The P4P program sought to engage various clinical departments through the appointment of Well-being Leads to ensure adoption, quality control, and sustainability. Built on the principles of providing support for peers in a nonevaluative, confidential, and psychologically safe manner, the P4P program sought to provide empathetic listening, supportive resources, and external referrals to knowledgeable experts. The P4P program also sought to build capacity in its Well-being Leads by providing them with the necessary knowledge and tools. To do so, a faculty-wide survey was implemented to identify different themes of focus that were prioritized by physician leaders when providing support. Identified priorities were formalized into a Royal College Continuing Professional Development–accredited skills training curriculum that covered empathetic listening; recognizing signs of mental distress; professionalism and ethics; unconscious bias; harassment and intimidation; and ethics, inclusion, and diversity. In addition to the training curriculum, Well-being Leads also attended monthly rounds for continued support, networking, and involvement in continued program development.

### Program Enrollment and Evaluation

In addition to a formal announcement of the program availability throughout the faculty, Well-being Leads were tasked with outreach and promotion of the program. Participation in the program were envisioned to be both formal and informal and followed the 7 basic steps to empathetic listening. Physicians seeking support could connect with their Well-being Leads by invitation, and a conversation using the empathic skills of opening, listening, reflecting, reframing, sense making, coping, and closing would occur in a nonjudgmental supportive environment.

The program was developed in March and launched in April 2020. Potential Well-being Leads were either identified by the Department Chair or through self-identification. Criteria for the attributes of Well-being Leads and participation outcomes are noted in Textbox 1. Skills training were conducted for over 30 divisional Well-being Leads within the Schulich School of Medicine and Dentistry to start. A second wave of training and promotion was launched in December 2021. The second wave included additional training conducted for new Well-being Leads, expanding the number of leads to 55. Well-being Leads training was conducted online via Moodle, a web-based, interactive self-learning platform. Once Well-being Leads were trained, they were deployed within their respective divisions as points of contact for program participants. To preserve the anonymity of program participants, the engagement and reach of the program was assessed indirectly through a survey of the Well-being Leads across divisions. Well-being Leads completed surveys documenting the number of encounters in the program via type of chat, reason for chat, referral (if identified), and the gender of participating peer.

**Criteria for identification and participation.**

**Attributes of Well-being Leads**

- Experience in providing peer support in the past
- Interest in physician well-being
- Background in education or research in physician burnout
- Alignment to personal values that suited the role
- Nonleadership role (*optional*)

**Participation Outcomes**

- Noted contribution as an academic activity toward promotion dossier
- Entry into academic curriculum vitae by the decanal office at the Schulich School of Medicine and Dentistry, Western University

## Results

### Reach of P4P Program

During the first wave, trained Well-being Leads reported 85 encounters with peers, whereas wave 2 saw an increase to 288 encounters. As of January 2023, reach has increased to 443 encounters and expanded into additional departments within the Schulich School of Medicine and Dentistry and regional and community networks within and outside of London, Ontario.

### Topics and Outcomes

The topics during wave 2, which are nonmutually exclusive, centered around mental well-being support (110/288, 38.2% of conversations), family issues (46/288, 16% of conversations), and COVID-19–related concerns (40/288, 13.9% of conversations). Moreover, 83% (239/288) of encounters required no further referral for additional help. Furthermore, 5.2% (15/288) were referred to the decanal office for support provided by the Assistant Dean, Faculty Wellbeing with consultant resources (ie, psychologist, therapist, etc) through Western University’s human resources, and another 5.2% (15/288) wished to pursue additional help privately.

## Discussion

### Principal Findings

Despite the high levels of mental distress, physicians continue to report low levels of engagement with the use of support services. Due to the elevated stress reported by physicians and the complexities surrounding professional issues and stigma, researchers have proposed solutions that are created for physicians by physicians [6]. In our paper, we documented the rapid development and deployment of a peer support solution implemented for physicians within the Schulich School of Medicine, Western University in London, Ontario, Canada.

The reach and uptake of the program over both waves reflect its continued expansion into 2023. The acceptability of the program is in part reflected in the organizational commitment and infrastructural support.

To support program deployment, a Well-being Executive Committee was formed as part of Schulich’s strategy to provide departments with support for expansion into other divisions, sections, and sites. In addition, the training was formalized through accreditation via Continuing Professional Development and Continuing Medical Education programs. These formalized processes, infrastructures, and training legitimized the program and facilitated subsequent participation across a range of programs within the larger institution. In addition, as the program evolved, new approaches were also utilized to engage physicians. Specific examples of engagement approaches included faculty-wide training offered to assess their own well-being and connect with peer leads, as well as offering monthly program support, newsletter, and individual rounding for Well-being Leads. Together, these efforts contribute to ongoing, collective, and cultural shifts in the organizational approach to mental health support.

### Conclusions and Future Direction

As of January 2023, the P4P program continues to grow and expand. The training curriculum continues to evolve based on feedback from Well-being Leads. Efforts are made to streamline participation online to reduce barriers of participation. The ease of access, confidential, and peer-based approach has been well accepted by the community of physicians. Indeed, the P4P program has gained interest from external entities. To date, the training curriculum has been utilized in Ontario, British Columbia, and Saskatchewan. With the information and processes identified through this paper, we urge future research to continue to examine processes of scaling up the P4P program for adoption in other organizations and communities to support physicians and other highly stressed workforces.

## Abbreviations

P4P: Peers for Peers

## Data Availability

Due to the ongoing nature of this peer support program, data may be available by request.
